# Adaptation to one perceived motion direction can generate multiple velocity aftereffects

**DOI:** 10.1167/jov.21.5.17

**Published:** 2021-05-18

**Authors:** Nikos Gekas, Pascal Mamassian

**Affiliations:** 1 School of Psychology, University of Nottingham, Nottingham, UK; 2 Laboratoire des Systèmes Perceptifs, Département d’études cognitives, École normale supérieure, PSL University, CNRS, Paris, France; 3 Laboratoire des Systèmes Perceptifs, Département d’études cognitives, École normale supérieure, PSL University, CNRS, Paris, France

**Keywords:** motion adaptation, multiple aftereffects, velocity, aperture problem

## Abstract

Sensory adaptation is a useful tool to identify the links between perceptual effects and neural mechanisms. Even though motion adaptation is one of the earliest and most documented aftereffects, few studies have investigated the perception of direction and speed of the aftereffect at the same time, that is the perceived velocity. Using a novel experimental paradigm, we simultaneously recorded the perceived direction and speed of leftward or rightward moving random dots before and after adaptation. For the adapting stimulus, we chose a horizontally-oriented broadband grating moving upward behind a circular aperture. Because of the aperture problem, the interpretation of this stimulus is ambiguous, being consistent with multiple velocities, and yet it is systematically perceived as moving at a single direction and speed. Here we ask whether the visual system adapts to the multiple velocities of the adaptor or to just the single perceived velocity. Our results show a strong repulsion aftereffect, away from the adapting velocity (downward and slower), that increases gradually for faster test stimuli as long as these stimuli include some velocities that match some of the ambiguous ones of the adaptor. In summary, the visual system seems to adapt to the multiple velocities of an ambiguous stimulus even though a single velocity is perceived. Our findings can be well described by a computational model that assumes a joint encoding of direction and speed and that includes an extended adaptation component that can represent all the possible velocities of the ambiguous stimulus.

## Introduction

The motion of a one-dimensional visual feature seen through a small aperture, such as the small receptive field of a velocity tuned channel (Marr & Ullman, 1981), is ambiguous. This is referred to as the aperture problem (Wallach, 1935; Würger, Shapley, & Rubin, 1996). For example, the motion of a horizontal grating moving upward that is seen through an aperture is compatible with all the directions that include an upward component, albeit at faster speeds. Even though humans resolve the perceptual ambiguity towards a direction that is perpendicular to the feature (e.g., Montagnini, Mamassian, Perrinet, Castet, & Masson, 2007), neurons in the early part of the visual system that are sensitive to other velocities should still be activated by the input stimulus. Although it may appear as a straightforward question, it is not clear how the visual system responds to prolonged exposure (adaptation) to such a stimulus. Does the visual system adapt to the single speed and direction of the way the stimulus is perceived, or to the multiple speeds and directions that the stimulus is consistent with because of the aperture problem?

Visual adaptation produces perceptual aftereffects that have been extensively documented over the last decades and provides a glimpse into the properties of the underlying neural mechanisms of perception. For example, prolonged exposure to a motion stimulus leads to strong, illusory perceptual biases in the perceived velocity (speed and direction) of static (“waterfall illusion,” Thompson, 1880) and moving stimuli (Clifford & Wenderoth, 1999; Ledgeway & Smith, 1997; Schrater & Simoncelli, 1998; Stocker & Simoncelli, 2009; Smith, 1985, Smith & Edgar, 1994, Thompson, 1981). Motion aftereffects (Anstis, Verstraten, & Mather, 1998) have been studied using various methods including physiological, psychophysical, and computational methods. However, explaining the different adaptation-induced biases under the same framework is not straightforward.

In this report, we are interested in adaptation effects for both speed and direction. There are very few studies that have investigated these two motion components at the same time (for notable exceptions, see Schrater & Simoncelli, 1998; Stocker & Simoncelli; 2009). Whether direction and speed are independently or jointly processed is still a matter of debate. While there is evidence that direction and speed can be dissociated in specific cases (Matthews & Qian, 1999; Matthews, Luber, Qian, & Lisanby, 2001; Saffell & Matthews, 2003; Curran & Benton, 2006), a systematic investigation of speed discrimination judgments at different directions showed strong interactions between direction and speed processing (Manning, Thomas, & Braddick, 2018). Stocker and Simoncelli (2009) proposed two isomorphic adaptation mechanisms that can explain motion aftereffects at a wide range of post-adaptation stimulus velocities, including zero velocities. One of the mechanisms is assumed to be nondirectional and consistent with mechanistic models of motion perception (“ratio models,” Perrone & Thiele, 2002; Hammett, Champion, Morland, & Thompson, 2005). In ratio models, the relative responses of low- and high-pass temporal frequency channels are compared to estimate stimulus speed but not direction. The output of the first mechanism provides input to a second mechanism of velocity tuned channels representing direction-selective neurons in cortical areas V1 or MT. This second mechanism assumes that direction and speed are jointly encoded as a vector entity.

In this report, we investigate whether the visual system adapts to one or multiple velocities of an ambiguous stimulus. Previous studies that have looked at simultaneous adaptation to two motion directions have suggested the possibility of adaptation at a level where directions are integrated (Riggs & Day, 1980; Verstraten, Fredericksen, & Van De Grind, 1994) or that when global visual representations are constructed, weak and inconsistent local signals are discarded (Kanai, Paffen, Gerbino, & Verstraten, 2004). On the other hand, it has been shown that adaptation to bi-stable (horizontal or vertical) ambiguous motion produces similar levels of adaptation to perceived as well as unperceived but possible motion directions (Hock, Schöner, & Hochstein, 1996). Here, we present a novel experimental paradigm to simultaneously measure the perceived direction and speed of stimuli moving at various velocities before and after adaptation. For the adapting stimulus, we use a one-dimensional grating moving upwards. For the test stimuli, we use a random dot kinematogram that moved either in a single direction that is parallel to the orientation of the adapting stimulus, or in multiple directions around that same direction of motion. In this latter case, the motion directions are controlled such that some of them are consistent with the ambiguous ones of the adapting stimulus. We hypothesized that adaptation to an ambiguous stimulus should affect not only the perpendicular velocity, which is the one that is perceived, but also all possible velocities of the adaptor's motion. This should produce an asymmetry in the adaptation of velocity channels; velocities with directions away from the perceived adapted direction should be affected only for speeds faster than the adapted speed but not for slower speeds. If speed and direction are encoded jointly by the visual system, we should be able to measure the differences in the strength of the motion aftereffect produced by the asymmetrical adaptation to these directions at faster speeds.

We find that, for stimuli composed of multiple directions of motion but constant speed, the motion aftereffect is significantly stronger for speeds that are faster than the adapting speed, and this effect increases with stimulus speed. On the other hand, for stimuli that have a unique direction of motion, we find that the strength of the motion aftereffect was uniform across speeds. We then present a computational model inspired by the proposal of Stocker and Simoncelli (2009) to incorporate both a nondirectional and a directional adaptation stage, and we show that both are necessary to explain our experimental findings. Our work suggests that observers adapt to the stimulus and not just the percept and provides further proof that direction and speed are jointly encoded in the visual cortex.

## Methods

### Participants

Ten adult human observers (five female) took part in the experiment, and they all had normal or corrected-to-normal vision. All participants were naive with regard to the purpose of the study, and they gave informed written consent in accordance with the local ethics committee and the Declaration of Helsinki.

### Stimuli

**Figure 1.**
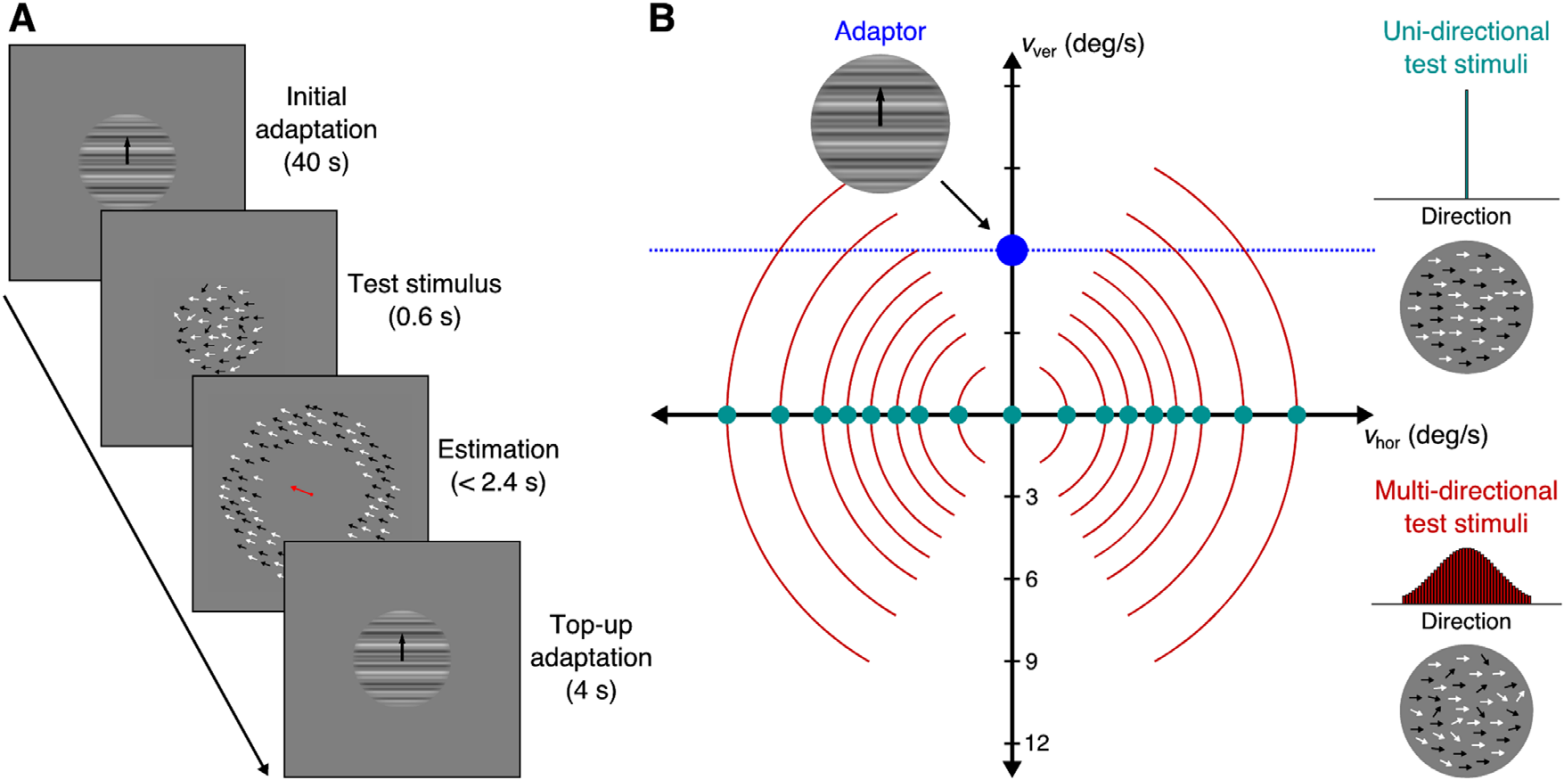
Experimental procedure. (A) After initial adaptation to an upward moving grating, a test RDK was presented to participants. After presentation, participants were asked to match the velocity of a ring of dots to the velocity of the test stimulus by freely moving a computer mouse that controlled a bar that extended from the center of the screen. The orientation and length of the bar correlated with the direction and speed of the stimulus’ motion. (B) The adapting stimuli were spatial broadband drifting gratings moving upward at 6°/s with phases randomized on each trial. The test stimuli moved rightward or leftward at eight different speeds or could be stationary. Teal dots indicate the unidirectional stimuli for which all dots moved in the same direction. Red arcs indicate the multidirectional stimuli for which dots had a fixed set of directions. The horizontal blue dotted line indicates the infinite possible velocities of the adapting stimulus due to the aperture problem. The red arcs at fast speeds intersect with the blue line, while they do not for slow speeds.

The adapting stimuli were high contrast (80%) spatial broadband drifting gratings (frequency range from 1/3 cycles/deg to 2 cycles/deg) moving upward at 6°/s with phases randomized on each trial. The adapting stimuli were shown inside a circular aperture of 10° in diameter. The test stimuli consisted of two types, random dot kinematograms (RDK) that moved coherently in one direction (*unidirectional*), and RDK that moved over a range of directions (*multidirectional*). The stimuli moved at eight different speeds (2°/s, 3.4°/s, 4.2°/s, 5.2°/s, 6°/s, 6.9°/s, 8.4°/s, and 10.5°/s) or were stationary (0°/s) (Figure 1B). The test stimuli were also shown inside a circular aperture of 10° in diameter. Each RDK had a contrast of 80%, density of 4 dots/deg^2^, and dot diameter of 0.1°. For the unidirectional stimuli, each dot moved at the same speed and direction (leftward or rightward, teal dots in Figure 1B), whereas for the multidirectional stimuli, each dot moved at the same speed but at a direction selected from a fixed set of directions (red arcs in Figure 1B). This set followed a normal distribution with mean at the central direction (leftward or rightward) and standard deviation of 30° and discretized at values ranging from −60° to 60° from the central direction in 3° steps. Thus a total of 41 possible directions with probabilities dropping from 4.16% at the center (0°) to 0.56% at the edges (±60°).

### Experimental procedure

The experimental procedure is illustrated in Figure 1A. An adapting stimulus was presented at fixation for 40 seconds. After the initial adaptation, the test stimulus was presented at fixation for 600 ms. Then, participants were presented with a bar extending from the center of the screen and a field of dots in a ring (the inner diameter was 10° and the outer 20°) outside the central area of presentation moving at the same velocity. Participants were able to manually alter the velocity of the ring of dots by moving the mouse. The orientation and length of the bar matched the direction and speed of the matching dots, respectively. Participants were asked to match the velocity of the ring stimulus to that of the test stimulus. When they were satisfied with their estimate, participants would need to click the mouse button to finish the trial. If they had not clicked the button before 2.4 seconds elapsed, their estimate was not saved, and the same trial was repeated at the end of the block. At the start of each new trial, there was a top-up adaptation for 4 seconds. An example trial showing the procedure along with examples of adaptor, test, and matching stimuli can be seen in Supplementary Movie S1. All stimuli were generated using the Matlab programming language with the psychophysics toolbox (MathWorks, Inc., Natick, MA, USA; Brainard, 1997) and displayed on a 21″ CRT monitor with a resolution of 1280 × 960 pixels at 100 Hz. Participants viewed the display in a darkened room at a viewing distance of 60 cm, and a chin rest was used to maintain a constant head location and viewing distance.

The test stimuli were presented in blocks of 40 trials, two trials per speed condition plus six trials with random speeds and directions slightly above or below leftward or rightward. These random trials were added to introduce ambiguity in the stimulus’ motion direction. Participants did three blocks for each type of test stimulus, first without adaptation, and then with adaptation in a single session. In total, there were six trials per participant for each of the 68 different conditions. At the beginning of the session, participants did at least 100 practice trials to get familiar with the task. In those trials, the speed and direction of test stimuli were randomized across the whole range of possible velocities and feedback was provided at the end of the trial in the form of a green Gaussian blob that indicated the correct speed and direction of the presented dots along with the participant's estimation as a red small dot. No adapting stimuli were shown during training. During the main part of the experiment, no feedback was given to participants.

Similar to Stocker and Simoncelli (2009), we are using a circular representation of speed *v*′ = 2arctan(*v*/*r*_0_), where *v* is the original speed in deg/s, *v*′ is the transformed speed, and *r*_0_ = 10. This closed space allows us to use a representation that is similar to a normalized logarithm but with the added benefit that it includes zero speed. The same space was used during participants’ estimation of the stimulus’ velocity, that is, the length of the estimation bar did not have a linear relation with speed. We run pilot sessions with both a linear and nonlinear relation, and we found that a nonlinear relation appears more natural to participants and better balances motor errors across speeds.

### Computational model

We implemented a neural model to simulate the effect of adapting to an ambiguous moving stimulus. The model is based on the one proposed by Stocker and Simoncelli (2009). Conceptually the models are very similar and any differences apply at the implementation level. As Stocker and Simoncelli do not describe their model in full detail, we cannot directly compare the two models, and we made choices of parameters (e.g., for the nondirectional and directional gain profiles) that best described our experimental results.

The network is composed of velocity channels φ, which are represented by bivariate Gaussian distributions:

(1) $$\varphi_{i}\left(x,y\right)∼\alpha N\left(\mu ,\Sigma \right),$$

where µ = [*x*0_*i*_, *y*0_*i*_] is the central horizontal (*x*0_*i*_) and vertical (*y*0_*i*_) velocity of the channel,

$$\Sigma =\left[\begin{array}{cc} \sigma_{\varphi }^{2} & 0\\ 0 & \sigma_{\varphi }^{2} \end{array}\right]$$

is the spread of the channel, and *α* is the maximum activity (“firing rate”) of the channel. Channels are homogeneously distributed in the horizontal-vertical closed velocity space, from −3 to 3 in units determined by the “arctan” transform defined above, in steps of 0.25 units for a total of 625 channels. As an illustration of the unit used for speed, 3 corresponds to 141°/sec whereas 1 to 5.46°/sec. A cartoon example of the network grid is shown in Figure 3A.

Adaptation to a stimulus is simulated in two stages. First, there is a nondirectional gain reduction that affects only fast or slow speed channels depending on the speed of the adapting stimulus. For slow speed adaptation, the gain reduction is expressed as a bivariate Gaussian function $r_{slow\_speed}∼w_{st1}N\left(0,\Sigma \right)$, where *w*_*st*1_ is the strength of the gain reduction and

$$\Sigma =\left[\begin{array}{cc} \sigma_{st1}^{2} & 0\\ 0 & \sigma_{st1}^{2} \end{array}\right],$$

and for the fast speed adaptation it is expressed as $r_{fast\_speed}=\max\left(r_{slow\_speed}\right)-\;r_{slow\_speed}$. After the nondirectional stage with fast speed adaptation, each channel's new activity profile is calculated as

(2) $$\varphi_{i}^{st1}=\varphi_{i}\left(1-r_{fast\_speed}\left(x0_{i},y0_{i}\right)\right),$$

The second stage is a directional gain reduction centered at the adapted velocity. This is expressed as $r_{directional}∼w_{st2}N\left(0,\Sigma \right)$, where *w*_*st*2_ is the strength of the gain reduction and

$$\Sigma =\left[\begin{array}{cc} \beta \sigma_{st2}^{2} & 0\\ 0 & \sigma_{st2}^{2} \end{array}\right].$$

Note the additional *β* parameter for the horizontal variance. We will see below that this parameter is chosen to be very large, thereby creating a directional adaptation that applies to all potential velocities of the ambiguous stimulus (blue horizontal line in Figure 1B). After the directional stage, each channel's new activity profile is calculated as

(3) $$\varphi_{i}^{st2}=\varphi_{i}^{st1}\left(1-r_{directional}\left(x0_{i},y0_{i}\right)\right),$$

There are two types of input stimuli. The uni-directional dots are assumed to be a bivariate Gaussian centered at the stimulus physical velocity and variance

$$\Sigma =\left[\begin{array}{cc} \sigma_{stimulus}^{2} & 0\\ 0 & \sigma_{stimulus}^{2} \end{array}\right].$$

The multidirectional dots are assumed to be a weighted sum of similar bivariate Gaussians centered at the different velocities (41 fixed velocities from −60 to 60) of the physical multidirectional stimulus and the same standard deviation. The weights are the probabilities of each direction of the physical stimulus (41 fixed values following a normal distribution with σ = 30^*o*^).

Each channel produces a response *n* to each stimulus *s* with Poisson variability based on the original φ*_i_* or altered $\varphi_{i}^{st2}$ activity profile to simulate the perceived velocity before and after adaptation:

(4) $$n_{i}\left(s\right)∼Poisson\left(\varphi_{i}\left(s\right)\right)$$

The horizontal *v_x_*(*s*) and vertical *v_y_*(*s*) velocities are calculated from the population average, so that:

(5) $$v_{x}\left(s\right)=\frac{\sum_{i}x0_{i}n_{i}\left(s\right)}{\sum_{i}n_{i}\left(s\right)}\;and\;v_{y}\left(s\right)=\frac{\sum_{i}y0_{i}n_{i}\left(s\right)}{\sum_{i}n_{i}\left(s\right)}$$

The model is run for 1000 iterations to produce averaged curves of expected biases at different stimulus horizontal velocities. A summary of the model's parameters is shown in Table 1. Only four parameters were allowed to vary to fit the experimental data: *w*_*st*1_, σ_*st*1_, *w*_*st*2_, σ_*st*2_.

**Table 1.** Parameters of the model.

Symbol	Value	Description
*α*	22	Maximum activity of the velocity channel
*β*	32^2^	Gain reduction of orthogonal velocity channel
*σ_ϕ_*	0.1	Velocity channel tuning standard deviation
*w*_*st*1_	1.586	Strength of the first (nondirectional) gain reduction stage
***σ*** *_st_*_1_	0.6209	Standard deviation of the first gain reduction stage
*w*_*st*2_	56.88	Strength of the second (directional) gain reduction stage
***σ_st_***_2_	0.5509	Standard deviation of the second gain reduction stage
*σ_stimulus_*	0.3162	Standard deviation of the stimulus

Bolded parameters were allowed to vary to match the experimental data.

## Results

### Experimental data

**Figure 2.**
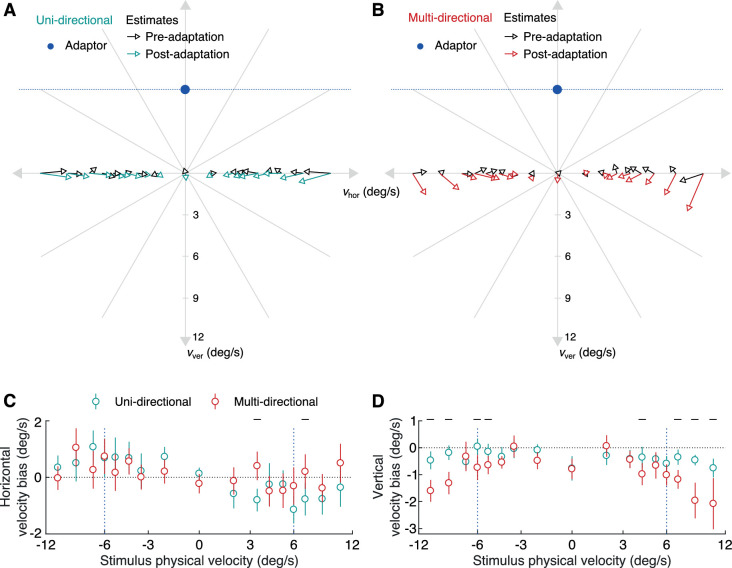
Estimated velocities before and after adaptation for the two types of test stimuli. (A and B) Estimated velocities are plotted as arrows for each stimulus physical velocity before (black) and after (colored) adaptation for unidirectional (A) and multidirectional (B) stimuli. The tail of each arrow indicates the physical velocity of the stimulus (or the average physical velocity for the multidirectional stimuli), whereas the head indicates the estimated velocity for that stimulus. Data points are averaged over all participants. The big blue dots indicate the physical velocity of the adaptor. (C and D) The differences between averaged estimates for horizontal (C) and vertical (D) velocities before and after adaptation are plotted for each physical stimulus velocity. Error bars indicate pooled standard error. The horizontal line above particular physical velocities indicates that there is a significant effect of stimulus type on estimation biases at *P* < 0.01 for these velocities.

Participants reported the perceived velocity of leftwards or rightwards moving dots before and after adapting to an upwards moving grating. Figure 2 shows these estimated velocities for each stimulus velocity, before (black) and after (colored) adaptation. Figure 2A shows the results obtained with the unidirectional test stimulus (teal color after adaptation), and Figure 2B the results with the multi-directional test (red color). The estimates are averaged over all participants’ responses so that each arrow in the figure corresponds to the average of 60 estimates. The tail of each arrow indicates the physical velocity of the stimulus and the head indicates the averaged estimates. For both types of stimuli and both before and after adaptation, participants tend to underestimate the speed of fast stimuli (long arrows pointing inward) and are more accurate (smaller arrows) for slow speeds. Looking at the postadaptation estimates (colored arrows), there is an apparent repulsion away from the adapted direction, with arrows for unidirectional and multidirectional pointing downward even for stationary stimuli. From a casual inspection, it appears that the effect is stronger for the multidirectional stimuli and the effect increases for fast speeds. We now analyze these effects more precisely.

The differences between the two types of stimuli can be better evaluated in Figures 2C and 2D. Participant estimates of direction and speed were decomposed into their horizontal and vertical velocity components. Figure 2C plots the averaged horizontal velocity biases between estimates before and after adaptation (i.e., $\Delta hor\_vel=hor\_vel_{post}-hor\_vel_{pre}$) for the unidirectional and multidirectional stimuli. Biases are positive for negative physical velocities and vice versa. The biases indicate an attraction towards slower speeds that is maximized around the adapting speed of 6°/s. We tested the significance of the differences between the two types of stimuli for each stimulus speed with a balanced two-way analysis of variance. We found a significant effect of stimulus type on estimation biases in only two of the 17 speeds at *P* < 0.01. These are indicated by the small horizontal lines above each stimulus speed in Figure 2C. For example, at 3.4°/sec rightward velocity, there was a significant effect of stimulus type on estimation biases (*F*(1100) = 21.11, *P* < 0.001)). Overall, the biases appear to follow a similar pattern for both types of stimuli.

Figure 2D plots the averaged vertical velocity biases between estimates before and after adaptation (i.e., $\Delta ver\_vel=ver\_vel_{post}-ver\_vel_{pre}$) for the unidirectional and multidirectional stimuli. The repulsive effect is present for both types and appears larger for the multi-directional stimuli. We again tested the significance of this effect. We found a significance effect of stimulus type on estimation biases in eight of the 17 speeds at *P* < 0.01. In particular, there is a significant effect for all but one speed faster than the adapted speed (five of six). For example, at the highest rightward speed of 10.5°/sec, there was a significant effect of stimulus type on estimation biases (*F*(1100) = 10.29, *P* = 0.002). For speeds slower than the adapted speed, only two of nine differences are significant at *P* < 0.01. Finally, of interest are the almost identical biases to stationary stimuli for both types of stimuli. Obviously, the two stimuli are identical because there is no movement, so the similarity suggests a quite robust effect. All responses by individual participants decomposed to their horizontal and vertical components are plotted in Supplementary Figures S1 and S2, respectively, where it can be seen that estimation behavior was consistent across participants.

### Model simulations

**Figure 3.**
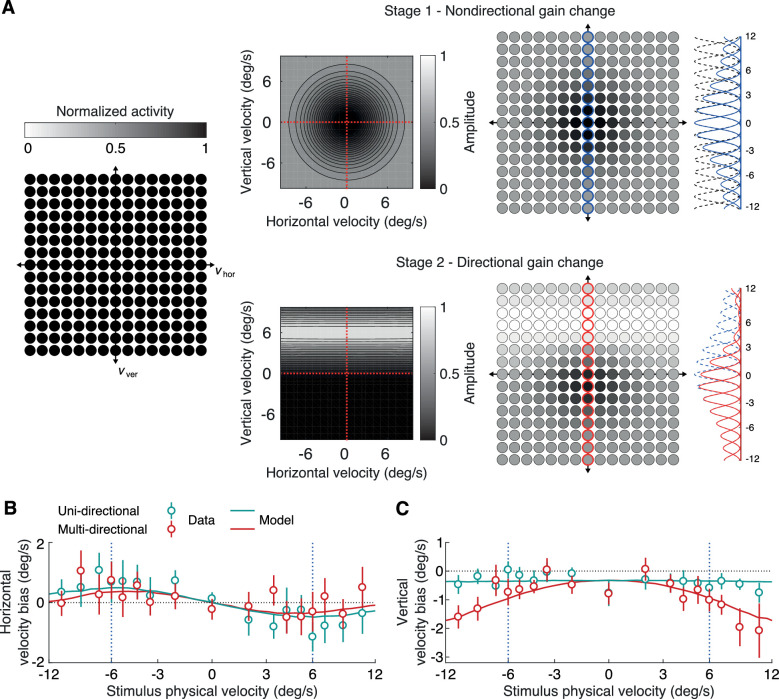
Model illustration and model fits. (A) The computational model designed to explain the adaptation effects consists of gain reduction at two stages. Left panel: Velocity channels are distributed uniformly along the horizontal and vertical velocity axis. The 15 × 15 gird of circles correspond to the sub set of the 125 central channels of the full model (25 × 25, 625 channels). The brightness of each disc indicates the maximum response of the channel (black is full maximal response). Before adaptation, all channel respond equally strongly. Top-right panel: Adaptation gain reduction profile for the nondirectional stage. After the first stage of adaptation, maximum responses are reduced for channels that encode fast speeds. One-dimensional slices along the vertical velocity axis for the mean response of each channel centered at zero horizontal velocity (blue circled channels). The black dashed and blue solid lines indicate the mean response of each channel before and after the gain reduction. Bottom-right panel: Adaptation gain reduction profile for the directional stage along with channel responses after the second stage of adaptation. One-dimensional slices for the same channels (red circled channels) before (blue dashed) and after (red solid) the second stage. (B and C) Biases for horizontal (B) and vertical (C) velocity estimates are plotted for each physical stimulus velocity. Open colored dots indicate experimental data and error bars indicate pooled standard error. The solid colored lines represent fits of the model for each stimulus physical velocity.

The experimental results seem to validate our hypothesis that the upwards velocities of the fast multidirectional stimuli are strongly affected by the adapting stimulus even though they are further away in velocity space. To explain our findings, we implemented a computational model of velocity channels and simulated the effect of adaptation to an upward moving stimulus (see Methods). Figure 3A shows the two stages of gain reduction to velocity channels. In the first nondirectional stage, channels that encode high speeds across the whole velocity space are strongly affected, whereas channels that encode slow speeds are less affected or not at all. We show the reduction in response to the central 125 channels as a change in their color (light shades indicate smaller response and dark shades larger response). We also plot the cross-section of the response profile of channels encoding zero horizontal velocity (blue and red circled channels in Figure 3A) to illustrate the change induced by the two stages after adaptation. The channels’ original tuning profiles are plotted in black dashed lines, and the new profiles after the nondirectional stage are plotted in blue solid lines. Channels that encode fast speeds are equally affected by the first stage independent of direction. In the second directional stage, channels that are close to the adapting velocity are affected more than channels that are further away. However, we propose that this effect applies equally to all possible velocities of the ambiguous adaptor. So, channels that encode the same vertical velocity as the adaptor but different horizontal velocities are equally affected. The channels’ new tuning profiles after the directional stage are plotted in red solid lines. Even a channel that encodes zero vertical and horizontal velocities is affected in the second stage.

**Figure 4.**
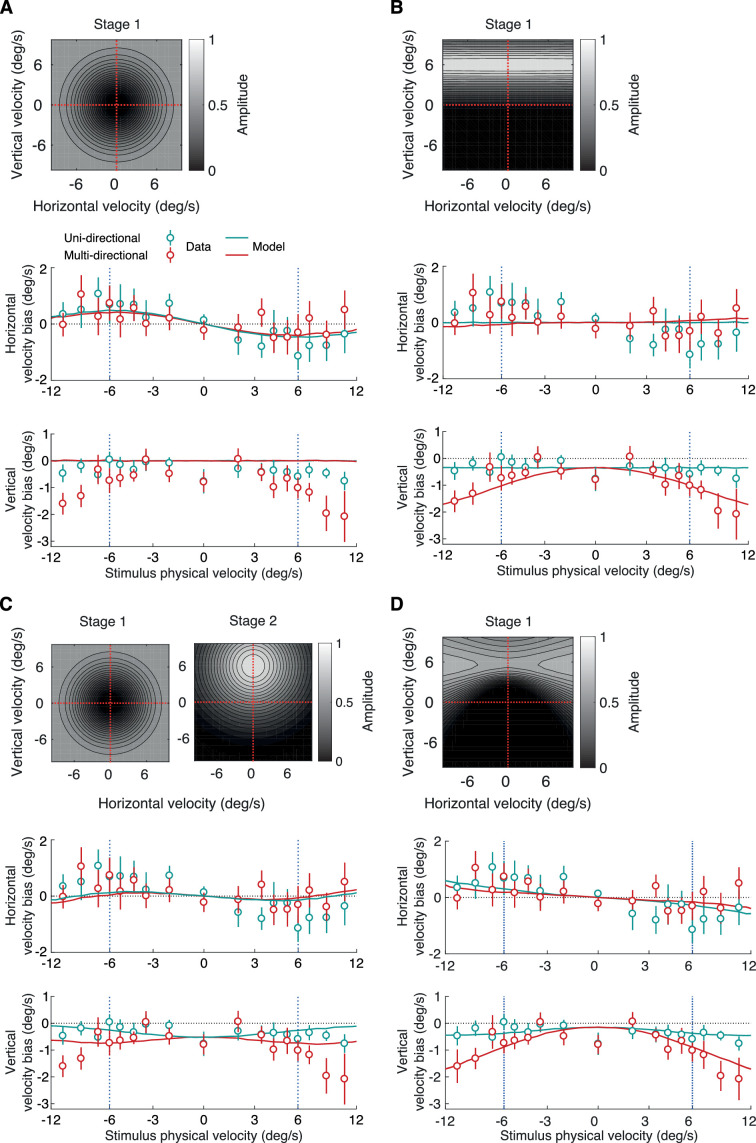
Model fits for different adaptation profiles. (A) Bias fits after applying only the nondirectional stage of adaptation. The model predicts horizontal velocity biases but not vertical. (B) Bias fits after applying only the directional stage of adaptation. The model predicts vertical velocity biases but not horizontal. (C) Bias fits after applying the nondirectional stage of adaptation and a modified directional stage that assumes that adaptation is limited to the perceived velocity of the adaptor. The model fails to predict the pattern of vertical velocity biases at fast speeds. (D) Bias fits after applying only a directional stage of adaptation with an extended profile over horizontal velocities. The model predicts vertical velocity biases but only weak horizontal biases that monotonically increase with stimulus speed.

Our goal with the proposed model is not to precisely predict the absolute speed estimates of participants but rather to explain the changes in speed estimates between before and after adaptation. Our preadaptation data present some biases toward slow speeds that may have a number of possible origins, including a sensory prior for slow speeds, a motor bias because of the reproduction method we used, or a response bias reflecting a regression to the mean. We assume that these biases are independent of the adaptation, thereby allowing us to focus on the differences in estimates between before and after adaptation. Figure 3B plots the experimental data along with the predictions of the model for the horizontal velocity biases. The model replicates the pattern of attractive biases toward slower speeds and the increase of the effect around the adapting speed. This pattern can only be explained by the nondirectional gain reduction stage because the directional stage is not affecting channels that encode fast horizontal velocities. Figures 4A and 4B show predictions of the model after applying only one of the two stages. If only the directional stage is applied (Figure 4B), no biases are predicted for horizontal velocities. Figure 3C plots the experimental data along with the predictions of the model for the vertical velocity biases. For the unidirectional stimuli, the model predicts a weak uniform bias independent of the stimulus’ horizontal velocity in strong agreement with the experimental data. For the multidirectional stimuli, the model predicts a similarly weak bias at slow horizontal velocities that increases along with the stimulus’ horizontal velocity. Again, the model predictions closely match the experimental data. If only the nondirectional stage is applied (Figure 4A), there are no biases predicted for vertical velocities. If a modified directional stage is applied that assumes that adaptation is limited to the perceived velocity of the adaptor (i.e., *β* = 1), the model fails to correctly predict the pattern of vertical velocity biases at fast speed (Figure 4C).

Finally, we tested a version of the model with only the directional stage but with an “extended” gain profile where the bandwidth of velocity channels increases with speed. We used the energy model by Simoncelli and Heeger (1998) to simulate different adaptation profiles of a broadband grating drifting upward. An example profile and the produced fits can be seen in Figure 4D. The model is able to accurately replicate the vertical biases found in the data and predict horizontal biases without the nondirectional stage. However, the pattern of these horizontal biases differs from the experimental data in that they monotonically increase with stimulus speed instead of peaking at the adapted speed and decreasing for faster speeds. In summary, we think that the two-stage model provides the most accurate description of the data from all the models we tested. Interestingly, the two-stage model predicts weaker biases at zero velocity than the experimental biases. This could hint toward an imbalance in the distribution of velocity channels encoding slower speeds in comparison to channels encoding faster speeds. Overall, the modeling work suggests that both stages of gain reduction are required to explain participants’ perceptual biases and that the directional gain reduction profile of the adapting stimulus extends into faster horizontal velocities, up to at least 10.5°/s, and potentially infinitely.

## Discussion

Because of the aperture problem, a drifting grating will activate multiple neurons sensitive to different combinations of directions and speeds. In this report, we have shown that when such a moving stimulus is presented, observers adapt to all the velocities consistent with this ambiguous stimulus. When tested with stimuli moving in a single direction, participants exhibited uniformly weak biases towards downward directions, but when tested with stimuli that contained multiple directions, they exhibited increasingly stronger downward biases for increasing speeds. Intuitively, this can be explained by looking at the expansion of a multidirectional stimulus in velocity space (Figure 1B). As the speed of the test stimulus increases, the arc that represents the velocities of the stimulus expands into space and eventually goes beyond the horizontal blue line that represents the possible velocities of the ambiguous adapting stimulus. Thus a larger part of the multidirectional stimulus is affected for fast stimuli than for slow. The fact that this pattern is not present in the unidirectional stimuli suggests that the effect is not caused merely by the change in the speed of the stimulus.

A number of studies have looked at the effects of motion adaptation at multiple levels of the visual hierarchy (Curran, Clifford, & Benton, 2006; Lee & Lu, 2012; Lee & Lu, 2014; Scarfe & Johnston, 2011; Vidnyánszky, Blaser, & Papathomas, 2002). Scarfe and Johnston (2011) showed that the direction of a motion aftereffect produced by ambiguous local motion signals is modified by global motion. They argue for an interaction between the coding of local and global motion through feedforward and feedback connections between directionally coded cells and cells with large receptive fields. Such an architecture would allow the visual system to balance the competing requirements of retaining local precision but also integrating information over space to encode global motion. Local motion signals carried by two-dimensional elements such as RDKs are presumably integrated over space in a vector-average fashion (Amano, Edwards, Badcock, & Nishida, 2009). Our findings suggest that local one-dimensional motion ambiguity is solved globally rather than locally (see also Rider, Nishida, & Johnston, 2016; Nishida, Kawabe, Sawayama, & Fukiage, 2018). If this were not the case, we would not have observed the stronger biases to the perceived motion of the faster multi-directional stimuli. Our experimental paradigm could be useful in the investigation of global motion adaptation. Smith, Scott-Samuel, and Singh (2000) have shown that even when adaptation at the local motion level should not produce a net aftereffect, there is a consistent aftereffect away from the stimulus’ global motion percept, suggesting that adaptation also occurs at a level of visual processing where global motion is represented. To extend our work in this direction, good candidates of adapting stimuli would be Type II plaids (Ferrera & Wilson, 1990) because their perceived motion direction deviates from the sum of the motion vectors of their components.

The ambiguous adapting stimuli were broadband drifting gratings, whereas the test stimuli were random dot kinematograms. Ideally, adapting and test stimuli should have the same spatiotemporal frequency spectra in order to minimize the dimensions in which the stimuli differ. However, it would be very difficult to precisely control the different velocities of a multi-directional test stimulus composed of multiple superimposed drifting gratings. It has been shown that integration of stimuli of multiple spatiotemporal frequencies can affect the perceived speed for stimuli that share the same veridical speed (Jogan & Stocker, 2015) or different speeds (Gekas, Meso, Masson, & Mamassian, 2017). Moreover, it has been suggested that adaptation does not only alter the visual sensitivity for the frequencies of the adapting stimulus but also for multiple other frequencies in an orderly pattern of gains and losses (Gepshtein, Lesmes, & Albright, 2013). In our model, we did not simulate the differences in sensitivity across spatiotemporal frequencies and our stimuli were broadband to encompass multiple frequencies. Future studies can create a more accurate representation of the underlying velocity tuned channels by using stimuli with well-defined spatiotemporal frequency content such as plaids or frequency-controlled broadband dynamic noise stimuli called Motion Clouds (Sanz-Leon, Vanzetta, Masson, & Perrinet, 2012; Gekas et al., 2017).

We implemented a computational model with two stages of adaptation. First, a nondirectional gain reduction is applied that affects fast speed channels. Second, a directional gain reduction is applied that affects channels close to the adapting velocities but extends infinitely into faster horizontal velocities at the same vertical velocity. To find signatures of the two mechanisms, both must be involved in the experimental task. In experiments where participants only report the speed or the direction of a stimulus, it is difficult to isolate the effects of these two potential mechanisms. Stocker and Simoncelli (2009) were able to design an experiment to isolate the nondirectional mechanism even though participants only reported the speed of the stimulus. Participants adapted to an upward moving stimulus and reported the perceived speed of grating stimuli moving leftward or rightward but not the perceived direction. With our experimental design, we were able to not only measure the speed bias at the orthogonal velocities, like Stocker and Simoncelli did, but also at the parallel velocities. This allowed us to identify signatures of both mechanisms simultaneously and the manipulation of the stimulus’ directional composition allowed us in turn to map the effect of the directional mechanism across the velocity space. Simulations showed that both mechanisms are required to explain the experimental results because the application of only one of the mechanisms cannot explain biases to estimated horizontal and vertical velocities.

Neurophysiological studies have found that neurons in the visual cortex change their response profiles after adaptation (Dragoi, Sharma, & Sur, 2000; Kohn & Movshon, 2003; Kohn & Movshon, 2004). These changes can include reduction in responsivity (Kohn, 2007), broadening of tuning widths (Dragoi et al., 2000), and shift of the preferred velocity away from the adaptor (Dragoi et al, 2000). For simplicity, we only modeled the reduction in responsivity, thus implementing a more parsimonious model with fewer free parameters. Still, we were able to accurately simulate participants’ biases for the two types of stimuli. We also found stronger than expected biases to static stimuli that suggested a potential asymmetry in the distribution of velocity channels towards slow speeds. Such asymmetries could potentially also explain the well-documented preference for slow speeds (Stocker & Simoncelli, 2006; Vintch & Gardner, 2014), and more specific direction-depended speed biases identified by Manning et al. (2018) and Moscatelli, La Scaleia, Zago, and Lacquaniti (2019). We believe that identifying the topography of the joint direction-speed space is a very promising direction for future research, and our experimental paradigm could prove a useful tool in accomplishing that.

## Supplementary Material

Supplement 1

Supplement 2

Supplement 3
