# Supplementary material for: Adaptation to one perceived motion direction can generate multiple velocity aftereffects
Source: J Vis. 2021 May 18;21(5):17. doi: 10.1167/jov.21.5.17 (PMC8142737; doi:10.1167/jov.21.5.17)
Supplement: Supplement 1 [file jovi-21-5-17_s001.pdf]

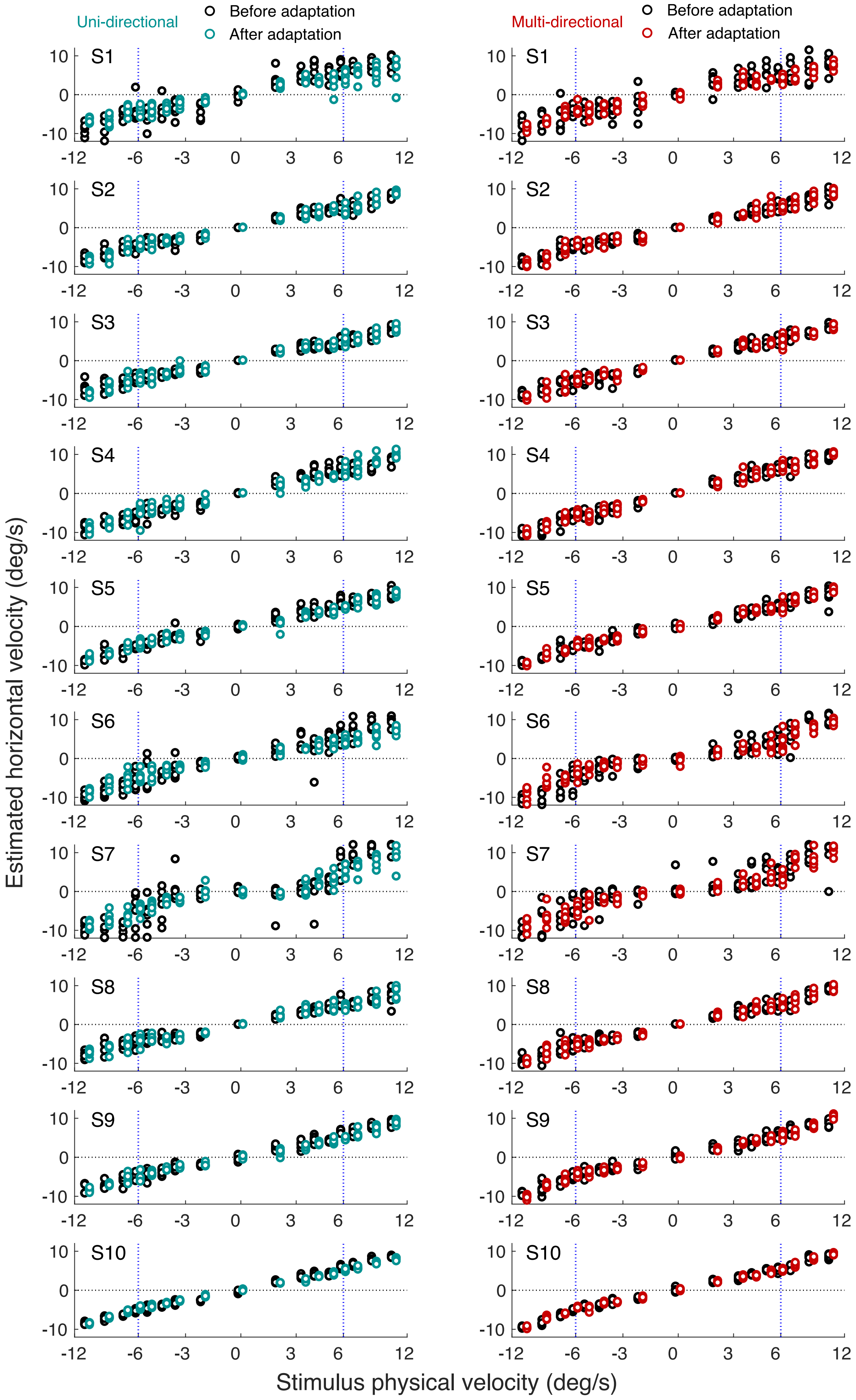

Uni-directional
Before adaptation
After adaptation
Multi-directional
Before adaptation
After adaptation
S1
S2
S3
S4
S5
S6
S7
S8
S9
S10
Estimated horizontal velocity (deg/s)
Stimulus physical velocity (deg/s)

Supplementary Figure S1. All estimated horizontal velocities are plotted for each stimulus physical velocity before (black) and after (colored) adaptation for uni- (left) and multi-directional (right) stimuli for all participants.
